# False Beliefs About ART Effectiveness, Side Effects and the Consequences of Non-retention and Non-adherence Among ART Patients in Livingstone, Zambia

**DOI:** 10.1007/s10461-012-0221-2

**Published:** 2012-06-20

**Authors:** Ikuma Nozaki, Mika Kuriyama, Pauline Manyepa, Matilda K. Zyambo, Kazuhiro Kakimoto, Till Bärnighausen

**Affiliations:** 1 Technical Advisor on Rural ART Services, Ministry of Health, Lusaka, Zambia; 2 Department of International Medical Cooperation, Japan National Center for Global Health and Medicine, 1-21-1, Toyama, Shinjuku, Tokyo 162-8655 Japan; 3 Department of Global Health and Population, Harvard School of Public Health, Boston, MA USA; 4 Livingstone, Zambia; 5 School of Nursing, University of the Sunshine Coast, Queensland, Austria; 6 Livingstone District Health Management Team, Livingstone, Zambia; 7 School of Nursing, Osaka Prefecture University, Osaka, Japan; 8 Africa Centre for Health and Population Studies, University of KwaZulu-Natal, Mtubatuba, South Africa

**Keywords:** Sub-Sahara Africa, Antiretroviral treatment, Knowledge, Beliefs

## Abstract

Beliefs about antiretroviral treatment (ART) are crucial for treatment success but not well documented in sub-Sahara African countries. We studied the frequency of false beliefs about ART in 389 ART patients in Livingstone, Zambia. Despite intensive pre-ART counseling, we find that more than half of the patients hold at least one false belief about ART effectiveness, side effects, or the consequences of ART non-retention or non-adherence. Commonly held false beliefs—e.g., pastors can cure HIV infection through prayer and ART can be stopped without harmful effects while taking immune-boosting herbs—are likely to decrease ART adherence and retention.

## Introduction

With an adult HIV prevalence of 14 % [1], Zambia is one of the countries in sub-Saharan Africa highly affected by the HIV epidemic. Antiretroviral treatment (ART) became available in the public sector in Zambia in 2002; the pace of ART scale-up accelerated rapidly in 2004 and 2005. By December 2009, more than a quarter of a million people were receiving ART in the country [2]. It is well documented that the success of ART depends crucially on patient retention in care and high levels of treatment adherence [3–6]. ART patients’ behavior, in turn, is likely to depend in part on beliefs about treatment effectiveness, side effects, and the consequences of non-retention and non-adherence [7, 8]. It is thus important that ART patients do not hold wrong beliefs about the treatment they receive. For this reason, ART initiation in many treatment programs in sub-Saharan Africa is preceded by several teaching sessions about ART [9, 10]. Only two previous studies in sub-Saharan Africa have reported beliefs about ART in ART patients [10, 11]. These studies took place in the early phases of ART scale-up in the respective countries (South Africa in 2002 [10] and Togo in 2005 [11]). Several years into the large-scale ART provision in sub-Saharan Africa, our understanding of the beliefs ART patients hold about their treatment is still limited.

Here, we report for the first time findings on the frequency of false beliefs about ART among ART patients several years after the start of the public-sector ART scale up in a sub-Saharan African country. Our study took place in primary healthcare centers around Livingstone, Zambia.

## Methods

This study was conducted in 2009 in the Livingstone District of Zambia, which is located at the border to Zimbabwe. The district consists of Livingstone City and surrounding peri-urban areas. The district has one general tertiary hospital and 13 primary healthcare centers the city. Of the 13 healthcare centers, 11 provide ART. The interviewer team for this study visited each of the 11 healthcare centers once on a randomly selected day over a 2-week period.

All ART patients aged 18 and above who came to receive ART at one of the 11 health centers during the day of the visit by the study interviewer team were asked to participate in face-to-face interviews. ART enrolment, the minimum age of 18, and ability to give informed consent were the only study inclusion criteria; we did not apply any exclusion criteria. Of 421 ART patients who visited a health center during one of the interview days, 403 patients consented to study participation (i.e., non-participation was less than 5 %). We excluded 14 (or 3 %) of the 403 study participants from our final sample, because they had at least one missing value for one of the variables used in the analysis. Our final sample thus included 389 of the total of 1,578 active ART patients in the 11 healthcare centers in the district. All patients in this sample provided informed, written consent to participate in the study. The interview included questions on socio-demographic variables and 10 true-or-false questions to evaluate different dimensions of ART knowledge (on ART effectiveness and side effects, ART retention, and ART adherence). We tested the questions used in this study by asking 25 patients and three health workers to tell us how they understood the question and what they would consider in answering it. We revised the initial questionnaire based on the results of this pilot study, replacing phrases and words that could be misunderstood with alternatives that the pilot study participants indicated did not carry the risk of misunderstanding.

Data were processed and analyzed in SPSS 17.0 for Macintosh. A multivariable logistic regression model was used to examine conditional associations of the socio-demographic variables (age, sex, education, electricity at home, family support for ART treatment, and duration of ART) with perfect knowledge (i.e., answering all ART knowledge questions correctly).

## Results

The median age of the respondents was 40 years (interquartile range (IQR) 32–46), and the median months on treatment was 23 (IQR 12–41). Of the 389 respondents, 5 % were male; 50 % had attained secondary-school level of education or higher; 70 % were currently not working; 27 % were living in a house with electricity; and 88 % received family support for ART treatment.

Overall, 56 % of respondents held at least one false belief about ART (Table 1). Of all respondents, 41 % held at least one false belief about ART effectiveness and side effects; 17 % held at least one false belief about ART retention; and 17 % held at least one false belief about ART adherence. Table 2 shows the results of regressing an indicator variable of perfect ART knowledge on age, sex, education, electricity at home, family support of ART taking, and duration of ART.

**Table 1:** Art beliefs

Statement	% (95 % CI) of patients who agreed with the false statement shown in the first column
Effectiveness and side effects	
Pastors can cure HIV by prayer	17 % (13–21 %)
HIV can be cured by ART*	17 % (13–21 %)
If you start ART you will die soon	3 % (2–5 %)
ARVs cannot cause side-effects such as vomiting, rash, pain in legs*	14 % (11–18 %)
Patients with at least on false belief about ART effectiveness and side effects	41 % (36–46 %)
Retention	
You do not have to take ARVs for the rest of your life	6 % (4–9 %)
You can stop taking ARVs while you are taking immune-boosting herbs	10 % (7–13 %)
You can stop ART after you regain health	4 % (2–7 %)
You can stop ART without consulting health workers if you have side effect	2 % (0–4 %)
Patients with at least one false belief about ART retention	17 % (13–21 %)
Adherence	
Missing doses of ARVs does not lead to disease progression*	8 % (5–11 %)
There is no risk of ARVs becoming ineffective in future if you stop taking ARVs	14 % (11–18 %)
Patients with at least one false belief about ART adherence	17 % (13–21 %)
At least one false belief	56 % (51–61 %)

*N* = 389, *ARV* antiretroviral drugs, *ART* antiretroviral treatment, *CI* confidence interval

* Questions from a questionnaire used previously in South Africa [10]

**Table 2:** Multivariable logistic regression analysis of factors associated with perfect ART knowledge

	Odds Ratio (95 % CI)	*P* value
Age		
≥40 years	1.44 (0.94–2.20)	0.095
<40 years	1	
Sex		
Male	0.84 (0.52–1.37)	0.491
Female	1	
Educational attainment		
Secondary or higher	1.64 (1.06–2.52)	0.025
Primary or lower	1	
Electricity at Home		
Yes	0.58 (0.35–0.95)	0.030
No	1	
Family support		
Yes	2.38 (1.19–4.76)	0.014
No	1	
Duration of ART		
0–12 months	0.73 (0.44–1.21)	0.217
13–24 months	0.54 (0.32–0.91)	0.020
≥24 months	1	

*ART* antiretroviral treatment, *CI* confidence interval

## Discussion

While 95 % of respondents correctly replied that they were aware that they would have to take medicine every day for life once they began ART, 17 % of participants falsely believed that HIV could be cured by ART. These two beliefs seem incompatible but were jointly held by 16 % of all respondents, suggesting fundamental misunderstandings of the functioning of ART even after the comprehensive ART counseling and education HIV patients receive preceding ART initiation. Another particularly worrisome false belief is that pastors can cure HIV infection through prayer (held by 17 % of participants), which suggests that these patients may at some point decide to discontinue medical treatment and seek cure through prayer. This hypothesis is supported by anecdotes that ART health workers and patients shared informally with members of the interview team recounting that some patients had stopped ART after attending prayer services promising to cure their HIV infection.

The percentage of participants who described at least one false belief relating to ART adherence was 17 %, a figure that seems high given that all of the respondents had previously participated in adherence counseling sessions and were asked about adherence during the routine ART clinic visits. Almost one in six respondents believed that ART could not cause side effects. Such a false belief is likely to lead to unnecessary health losses among ART patients, as patients who do not realize that their treatment may be the source of symptoms, may not report certain symptoms to health workers, thus limiting the workers' ability to intervene early and appropriately [12].

While we cannot rule out that some participants misunderstood a particular question—for instance, because they interpreted the word “cure” as meaning “absence of symptoms” rather than “elimination of HIV”—it seems unlikely that misunderstandings of question meanings were common because, as described above, we carefully tested the study questionnaire in a pilot study and revised the initial questions based on the pilot results to minimize the risk that particular words and phrases could be misunderstood.

A prior study in South Africa [10] found that 50 % of respondents believed that HIV can be cured by ART and 36 % believed that ART cannot cause side effects. The participants in our study were substantially less likely to hold these false beliefs (17 and 14 %, respectively). There are several possible reasons for this difference. For one, all participants in our study were on ART at the time of the interview, while in the South African study only 30 % of participants received ART [10]. This difference in sample composition might explain the differential in false beliefs: People receiving ART have first-hand experience with the treatment, and it is likely that they have received more ART-specific education than those not on ART. Furthermore, our study took place several years after the South African study and it is likely that over calendar time ART knowledge in the Southern African region has increased.

Our findings that the likelihood of perfect ART knowledge improves with increasing education and the presence of family support conform with our expectations. People with higher levels of general education are likely to have better access to information on ART and may have had more exposure to teaching about human biology and other areas of knowledge that are relevant to understanding the functioning of ART. People whose families support them in their ART treatment are likely to have more opportunities to examine and improve their understanding of the functioning of ART in discussions with others. Contrary to our expectation, the presence of electricity at home, a proxy for socioeconomic status, was negatively associated with perfect ART knowledge in multivariable regression. It is possible that this finding can be explained by a selection effect: people of lower socioeconomic status are likely to face higher hurdles to ART access, for instance, because they may find it difficult to pay for transport to the healthcare centers where ART is available. As a result, only those in this group who are particularly knowledgeable and motivated to receive ART may access the treatment. In contrast, people of higher socioeconomic status may access ART even if they are not very knowledgeable and motivated to receive ART, because they do not face substantial hurdles to access.

Our findings further suggest a u-shaped relationship between ART duration and knowledge. Such a relationship could be explained by interactions of several effects, such as health workers improving their skills in explaining the functioning of ART over time, patients increasingly seeking out alternative sources of information on treating HIV infection as they develop unpleasant side effects on ART, and increased drop-out of patients with imperfect ART knowledge. Future research needs to further elucidate the determinants of ART knowledge, in order to ensure that education and counseling of ART patients is effective and appropriate at different stages in the course of ART. It will also be important to understand in how far the quality and intensity of the current education and counseling in primary healthcare centers in Zambia can be increased.

Previous studies have found that beliefs about ART predict ART adherence [13, 14], but none of these studies examined the effect of beliefs on adherence within sub-Saharan Africa. Furthermore, no study has investigated the relationship between belief about ART and retention in HIV care. We find that a substantial proportion of patients believe that they do not have to take antiretroviral drugs (ARV) for the rest of their lives, and that they can stop taking ARV while taking immune-boosting herbs, when side effects occur, or after their health status has improved. Such beliefs are likely to increase the risk of treatment discontinuation. To inform decisions on how to optimally allocate limited budgets for ensuring the long-term success of ART in Zambia, future work should investigate how important ART knowledge is for retention and adherence relative to other factors, such as the transport and time costs of accessing ART, forgetfulness, and stigma.

## References

[1] UNAIDS. Report on the global AIDS epidemic 2010, Geneva. 2010. http://www.unaids.org/globalreport/Global_report.htm. Accessed 6 Feb 2011.

[2] WHO/UNAIDS/UNICEF. Towards universal access: scaling up priority HIV/AIDS interventions in the sector: progress report 2010. Geneva: WHO; 2010.

[3] Bangsberg DR, Perry S, Charlebois ED (2001). Non-adherence to highly active antiretroviral therapy predicts progression to AIDS. AIDS..

[4] Weiss L, French T, Finkelstein R, Waters M, Mukherjee R, Agins B (2003). HIV-related knowledge and adherence to HAART. AIDS Care..

[5] Goujard C, Bernard N, Sohier N (2003). Impact of a patient education program on adherence to HIV medication: a randomized clinical trial. J Acquir Immune Defic Syndr.

[6] Roberts KJ (2000). Barriers to and facilitators of HIV-positive patients’ adherence to antiretroviral treatment regimens. AIDS Patient Care STDS..

[7] Bärnighausen T, Chaiyachati K, Chimbindi N, Peoples A, Haberer J, Newell ML (2011). Interventions to increase antiretroviral adherence in sub-Saharan Africa: a systematic review of evaluation studies. Lancet Infect Dis.

[8] Finocchario-Kessler S, Catley D, Thomson D, Bradley-Ewing A, Berkley-Patton J, Goggin K. Patient communication tools to enhance ART adherence counseling in low and high resource settings. Patient Educ Couns. 2012. doi: 10.1016/j.pec.2012.03.020. 10.1016/j.pec.2012.03.020 PMC3462269 22575433

[9] Torpey KE, Kabaso ME, Mutale LN (2008). Adherence support workers: a way to address human resource constraints in antiretroviral treatment programs in the public health setting in Zambia. PLoS ONE.

[10] Nachega JB, Lehman DA, Hlatshwayo D (2005). HIV/AIDS and antiretroviral treatment knowledge, attitudes, beliefs, and practices in HIV-infected adults in Soweto, South Africa. J Acquir Immune Defic Syndr..

[11] Potchoo Y, Tchamdja K, Balogou A, Pitche VP, Guissou IP, Kassang EK (2010). Knowledge and adherence to antiretroviral therapy among adult people living with HIV/AIDS treated in the health care centers of the association “Espoir Vie Togo” in Togo, West Africa. BMC Clin Pharmacol..

[12] Sanjobo N, Frich JC, Fretheim A (2008). Barriers and facilitators to patients’ adherence to antiretroviral treatment in Zambia: a qualitative study. SAHARA J..

[13] Chakrapani V, Newman PA, Shunmugam M, Kurian AK, Dubrow R (2009). Barriers to free antiretroviral treatment access for female sex workers in Chennai. India AIDS Patient Care STDS..

[14] Ramchandani SR, Mehta SH, Saple DG (2007). Knowledge, attitudes, and practices of antiretroviral therapy among HIV-infected adults attending private and public clinics in India. AIDS Patient Care STDS..

